# From Inflammasome to Exosome—Does Extracellular Vesicle Secretion Constitute an Inflammasome-Dependent Immune Response?

**DOI:** 10.3389/fimmu.2018.02188

**Published:** 2018-09-25

**Authors:** Wojciech Cypryk, Tuula A. Nyman, Sampsa Matikainen

**Affiliations:** ^1^Centre of Molecular and Macromolecular Studies, Polish Academy of Sciences, Lodz, Poland; ^2^Department of Immunology, Institute of Clinical Medicine, University of Oslo and Rikshospitalet Oslo, Oslo, Norway; ^3^Division of Rheumatology, University of Helsinki and Helsinki University Hospital, Helsinki, Finland

**Keywords:** inflammasome, inflammation, extracellular vesicle (EV), signaling, EV

## Abstract

Inflammasomes are intracellular protein complexes of pattern recognition receptors and caspase-1, with essential functions in regulating inflammatory responses of macrophages and dendritic cells. The primary role of inflammasomes is to catalyze processing and secretion of pro-inflammatory cytokines IL-1β and IL-18. Recently, intracellular non-canonical inflammasome activation by caspases-4/5, which are also regulators of pyroptosis via processing gasdermin D, has been elucidated. Caspase-1, the effector protease of inflammasome complex, is also known to modulate secretion of large number of other proteins. Thereby, besides its known role in processing pro-inflammatory cytokines, the inflammasome turns into a universal regulator of protein secretion, which allows the danger-exposed cells to release various proteins in order to alert and guide neighboring cells. Majority of these proteins are not secreted through the conventional ER-Golgi secretory pathway. Instead, they are segregated in membrane-enclosed compartment and secreted in nanosized extracellular vesicles, which protect their cargo and guide it for delivery. Growing evidence indicates that inflammasome activity correlates with enhanced secretion of extracellular vesicles and modulation of their protein cargo. This inflammasome-driven unconventional, vesicle-mediated secretion of multitude of immunoregulatory proteins may constitute a novel paradigm in inflammatory responses. In this mini review we discuss the current knowledge and highlight unsolved questions about metabolic processes, signals, and mechanisms linking inflammasome activity with regulated extracellular vesicle secretion of proteins. Further investigations on this relationship may in the future help understanding the significance of extracellular vesicle secretion in inflammatory diseases such as atherosclerosis, gouty arthritis, asthma, Alzheimer's and many others.

## Introduction

Extracellular vesicles (EV) are a heterogeneous group of membrane-enclosed carriers of biomolecules, secreted by all mammalian cells. This umbrella term refers to the pool of all vesicles, ranging in size from 30 to over 1,000 nm, constituting of exosomes (derived from endosomal compartment), ectosomes (microvesicles budding from plasma membrane) and apoptotic bodies [fragments of apoptotic cells, (1)]. Secretion of EV by immune cells constitutes a conserved mechanism of release of biomolecules (notably proteins, bioactive lipids and nucleic acids) both as a “waste disposal” and for targeted intercellular signaling. The latter became a major focus of immunologists' research efforts in the past decade. In innate immunity, secretion of EV by leukocytes is a tightly regulated process, dependent on the activation state of the cells (2). During immune response, EV are selectively loaded with immune regulatory proteins, like cytokines, what makes them important messengers delivering targeted biological signals. The vesicular signaling now emerges as a critical component of innate immunity that orchestrates actions of multiple immune system cells in infectious and inflammatory diseases.

Inflammasomes are molecular structures of the innate immune system that are critical for both local and systemic inflammation. Among them canonical NOD-like receptor protein 3 (NLRP3) is the most studied and known to sense multiple microbial and endogenous danger signals. NLRP3 activation results in caspase-1-dependent processing and secretion of critical pro-inflammatory cytokines interleukin (IL)-1β and IL-18, as well as drives pro-inflammatory cell death (pyroptosis). More recently non-canonical, caspase-4/5-dependent inflammasome has been described. These novel caspases binding to intracellular LPS, directly regulate pyroptosis, and activate canonical NLRP3-mediated IL-1β and IL-18 processing. Caspases-4/5 are critical for development of endotoxic shock in mice (3).

EV secretion has long been linked with inflammation and various experimental models were applied to characterize the roles the EV play in the development and progression of inflammatory diseases. In particular, EV isolated from patient body fluids have been studied in search for disease biomarkers and early diagnosis possibilities of such inflammatory diseases as atherosclerosis (4–6), Alzheimer's disease (7–10), and rheumatoid arthritis (11). EV secreted during inflammatory response are thought to promote inflammation in endothelial cells, neutrophils, hepatocytes, macrophages, and monocytes via various mechanisms: delivery of inflammasome components and cytokines, activation of surface receptors, induction of expression of inflammatory mediators and others [reviewed recently in (12)].

Contrary to extensive documentation on biological functions of EV in inflammatory diseases, mechanistic explanations of how the inflammasome activity regulates secretion of EV and modulates their cargo and biological properties in inflammatory cells are vague. Use of high-throughput proteomic approaches has contributed substantially to our knowledge in these aspects and allowed to elucidate novel signals for EV secretion in inflammatory response. Using proteomics, we and others showed that canonical NLRP3 activators (calcium oxalate and monosodium urate (MSU) crystals, ATP, β-glucans, viral RNA) as well as non-canonical, caspase-4-dependent activation, induce secretion of EV and EV-associated proteins (13–18). In this mini review we discuss the current knowledge and highlight unsolved questions about signals, processes and mechanisms linking NLRP3 inflammasome activity with regulated EV-mediated secretion of pro-inflammatory cytokines and other immunoregulatory proteins.

## Multiple canonical NLRP3 activators induce EV secretion

### ATP

Extracellular ATP is an endogenous danger signal known to activate inflammatory response in innate immune cells including macrophages. The receptor for ATP is P2X purinoceptor 7 (P2X7), which is a transmembrane ATP-gated ion channel (19). Ligation of P2X7 results in strong activation of NLRP3 inflammasome (20). We have shown that ATP-induced NLRP3 inflammasome activation is followed by robust activation of EV-mediated protein secretion, including exosome release and membrane shedding in human macrophages (15). Interestingly, de Torre-Minguela et al. showed that P2X7-induced protein secretion does not depend on *de novo* protein synthesis (21). This observation suggests that activation of P2X7 ion channel causes rapid, probably non-specific sequestration of proteins at the vicinity of the plasma membrane and their release in shedding microvesicles. It is currently not clear if this process is inflammasome-dependent, as secretion of only part of the proteins was shown to be affected by caspase-1. Moreover, the study revealed multiple proteins whose secretion was caspase-1-dependent in M1 pro-inflammatory macrophages, but not M2, suggesting existence of more sophisticated, cell-specific regulatory mechanisms (21).

### Monosodium urate and β-glucans

Monosodium urate (MSU) and β-glucans are crystalline agents that activate NLRP3 inflammasome. MSU crystals have emerged as a causative factor for gouty arthritis through NLRP3 activation and following IL-1β secretion (22). β-glucans are the main components of fungal cell walls and they activate robust innate immune reaction. We have shown that both MSU crystals and β-glucans activate strong EV-mediated protein secretion in human macrophages (13, 14, 23). This protein secretion was dependent on the activity of Syk kinase and lysosomal proteases cathepsins, as well as caspase-1. This indicates that Syk kinase activity and lysosome damage, which both are involved in NLRP3 inflammasome activation (24), provide also a signal for the induction of EV secretion upon recognition of crystalline substances damaging lysosomes. These results also suggested that NLRP3 activity is required for MSU- and β-glucan-induced EV-mediated protein secretion.

### Nigericin

Nigericin is a microbial toxin derived from *Streptomyces hygroscopicus*, which has the ability to induce P2X7-independent potassium efflux and following activation of the NLRP3 inflammasome. Zhang and colleagues studied the effect of LPS and nigericin on EV secretion in mouse bone marrow-derived macrophages. LPS alone was not sufficient to induce substantial EV protein secretion as compared with LPS priming followed by nigericin stimulation (25). Thus, EV secretion may constitute a secondary effect of NLRP3 activity. Of note, proteomic analysis identified IL-1β and NLRP3 in the EV from LPS/nigericin-treated cells (25), adding EV-mediated secretory route to previous observations of secretion of inflammasome components during inflammatory response (22). Functionally, these EV were able to induce NF-κB signaling in recipient cells, suggesting that EV-mediated transport of proteins (and possibly other biomolecules) serves as a major intercellular signaling event in coordination of immune response (25).

## Non-canonical caspase-4/5 inflammasome activates EV secretion

The discovery that human caspases-4/5 (caspase-11 in mice) are novel pattern recognition receptors which directly recognize intracellular LPS fundamentally changed the understanding of inflammasome responses (3, 26–33). It was shown that caspases-4/5 activate NLRP3 inflammasome and processing of IL-1β and IL-18. Non-canonical caspases-4/5 also mediate cleavage of gasdermin D, yielding an N-terminal polypeptide which inserts into the cellular membrane and oligomerizes, forming a pore and inducing subsequent pyroptosis independently of NLRP3 (33, 34). Very recent data shows that gasdermin D also has a pyroptosis-independent function in phagocytic cells: it mediates IL-1β secretion in living macrophages which undergo “hyperactivation” in response to bacteria and oxidized lipids (35). It was speculated that pyroptosis-independent IL-1β release may be a result of weak or modest activation of NLRP3 inflammasome, contrary to strong activation which triggers pyroptotic death (36).

Currently very limited data concerning EV secretion associated with non-canonical inflammasome response is available. In our recent study, we utilized label-free quantitative proteomics to characterize global protein secretion in response to non-canonical caspase-4/5 inflammasome activation in human macrophages (17). We demonstrated that non-canonical caspase-4/5 inflammasome activates robust EV-mediated protein secretion in human macrophages. Caspase-4 inhibitor blocked both IL-18 and EV secretion in macrophages in response to intracellular LPS treatment (17). However, it was not studied if NLRP3 inflammasome (which is also activated downstream of non-canonical caspases) is involved in the activation of EV-mediated protein secretion following intracellular LPS delivery. The role of gasdermin D in generation and secretion of EV is likewise unknown.

## The role of EV in NLRP3-mediated IL-1β release

Neither the 31kDa precursor nor mature 17 kDa form of IL-1β carry an N-terminal signal sequence. Upon processing by the inflammasome, mature IL-1β is targeted for release via poorly understood secretory route. Numerous mechanisms have been proposed to mediate IL-1β release, including homeostatic processes, like secretory autophagy (37–40) and lysosome exocytosis (41), as well as radical processes, resulting in cell death: pyroptosis (31) and necrosis (42). It has been suggested that rather than being secreted by one tightly controlled mechanism, the cytokine is exported in multiple ways simultaneously (43). Recent discoveries demonstrated that IL-1β secretion proceeds through gasdermin D membrane pores (31, 33, 35, 44), however it is still unclear how mature cytokine is exported through the pore. It seems that gasdermin D pore-mediated release is a primary secretory route for mature IL-1β secretion at initial stages of inflammatory response, as *Gsdmd*^−/−^ mouse macrophages secrete drastically reduced amounts of the cytokine upon canonical and non-canonical inflammasome stimulations (45). However, as gasdermin D-deficient mutants are resistant to pyroptosis but not apoptosis, it seems plausible that additional death-related mechanisms are involved in IL-1β secretion. For example, one study argued that secretion of mature IL-1β is a result of necrotic cell death, as THP1 macrophages that survived nigericin treatment retained the cytokine, contrary to dying cells (42). For more detailed perspectives on IL-1β processing and secretion the reader is referred to recent detailed reviews (40, 46).

Secretion of EV constitutes also an important auxiliary mean of IL-1β export. Both pro-form and mature IL-1β and IL-18 have been also observed in shedding microvesicles released from cells upon NLRP3 activation through distinct pathways (14, 47–49). It has been suggested that sequestration of activated inflammasome complex together with IL-1β and IL-18 precursors and their secretion on EV may precede the inflammasome activation within the lumen of the vesicle, thereby proposing an attractive explanation for observing both immature and mature cytokine forms present in EV (50). However, despite these and a small number of other studies identifying the presence of IL-1β in association with vesicles, the EV have not been fully elucidated as a secretory mechanism of IL-1β and IL-18.

## Mechanistic links between NLRP3 activity and EV secretion

### Transmembrane calcium/potassium fluxes

Changing transmembrane ionic balance of calcium and potassium is a critical signal for canonical NLRP3 activation (51–53). Potassium efflux and concomitant calcium influx is triggered by ionophores [streptolysin O (54), nigericin (55), maitotoxin (20)], as well as activators of P2X7 receptor (56), which are known for their rapid inflammasome activation. NLRP3 inflammasome is also activated by intracellular release of calcium from lysosomal compartment (55, 57).

Calcium influx is also a promoter of EV secretion (58). Calcium influx activates calcium-dependent floppase and scramblase, which are involved in exposing phosphatidylserine and phosphatidyethanolamine on the outer plasma membrane sheet, causing outward budding of the membrane. Subsequent reorganization of the cytoskeleton facilitates pinching off of the forming microvesicle (59).

Calcium (Ca^2+^) ions are important for cellular signaling, as once they enter the cytosol of the cytoplasm they regulate the activity of many enzymes and proteins. Calpains are cytosolic calcium-sensitive enzymes, involved in processing of many cytoskeletal proteins. They are also important for secretion of EV (60, 61). Crespin et al., showed that inhibition of calpains reduces microvesicle shedding resulting from calcium ionophore treatment in platelets (62). In our study we further explored the ATP-mediated inflammasome activation and EV secretion, showing that activation of calpains may be the missing link between P2X7-mediated ionic fluxes and inflammasome activation. In human macrophages, calpain inhibition supresses inflammasome activation and IL-1β secretion upon ATP and β-glucan treatment, but surprisingly not nigericin, a P2X7-independent potassium ionophore. It also completely abrogates ATP-induced EV secretion (15). This suggests that in macrophage P2X7-NLRP3 axis, calpain activation by calcium influx is required for both inflammasome activity and EV secretion, while for P2X7-independent ionophores calpain function may be dispensable.

### Lysosomal damage

Lysosome destabilization occurring during phagocytosis and endocytosis of crystalline particles is another well-established trigger of NLRP3 activation. These stimuli include cholesterol crystals (63, 64), MSU (22), calcium phosphate (65), and particulate β-glucans (66). During lysosomal membrane destabilization two major events have been demonstrated to play major role in triggering inflammasome assembly: leakage of lysosomal cathepsins, and potassium efflux.

Interestingly, we have also demonstrated that cathepsins play role in secretion of EV-associated proteins in the course of TLR3-mediated double-stranded RNA recognition in human macrophages in a model of viral infection (67). However whether EV secretion is modulated by cathepsin inhibition was not directly addressed. More recently, another group evaluated the roles of cathepsins in inflammasome activation, showing that neither widely used cathepsin inhibitor Ca-074-Me, nor alternative K777, are specific antagonists of a single cathepsin (68). Therefore the link between lysosome damage-induced NLRP3 activation and EV secretion is rather a combined effect of different cathepsins activity, whose roles need further elucidation. This also remains to be confirmed e.g., by using selective NLRP3 inhibitors. Concomitantly, the involvement of Syk and lysosomal cathepsins in activation of EV secretion needs also to be verified for other particulate NLRP3 activators like alum, asbestos or silica.

### Caspase-1 activity

Caspase-1, beyond its well-documented pro-IL-1β and pro-IL-18-processing functions, is also involved in secretion of other leaderless proteins. Keller and colleagues demonstrated that UVB irradiation-activated keratinocytes secrete over 900 proteins, of which secretion of 77 was significantly modulated in the presence of caspase-1 inhibitor YVAD (69). Interestingly, only a small number of these proteins are known to be direct caspase-1 targets (70). Many of them are also known to be secreted via EV. This revealed that caspase-1 may be directly involved in control of EV secretion and loading (70).

Moreover, upon discovering that inflammasome complexes can be secreted in EV, it has been proposed that caspase-1 activation is critical for sequestration of ASC specks and other inflammasome components in vesicular compartment for secretion and delivery to neighboring cells. Uptake of such “inflammasome-EV” is perceived as a mean of local propagation of inflammatory response (71, 72).

In contrast, a study published by Qu et al. argued against the critical role of caspase-1 in P2X7-mediated activation of EV secretion. The authors suggested that exosome secretion is a consequence of NLRP3 and ASC activity, while caspase-1 was dispensable (73). However, this study was performed on murine bone marrow-derived macrophages and dendritic cells and the EV secretion was mainly assessed by immunoblotting of MHCII, which is not a classical exosomal/EV marker, with lack of other validation methods. Corroborating this data, our study also indicated that caspase-1 inhibition by Z-YVAD-FMK was insufficient to prevent EV-mediated protein secretion induced by ATP in human macrophages (15).

## Conclusions and perspectives

Secretion of EV is currently perceived as a fundamental mode of intercellular communication, having impact on cell-to-cell delivery of proteins, lipids and nucleic acids. Circulating EV form a signaling network of the immune system, where various cells in blood and tissues exchange EV which coordinate homeostasis and alert the immune system upon sensing the danger. Growing number of studies now support the view that human phagocytic cells modulate secretion kinetics and cargo of EV during inflammatory response. There is strong evidence that EV secretion correlates with inflammasome activity (2, 13–18, 23, 25) and that the “inflammasome-induced” EV can activate inflammatory responses in recipient cells (25). Therefore activation of EV secretion via different mechanisms is possibly a universal outcome of inflammasome activity with evolutionary determined immune signaling.

**Figure 1:**
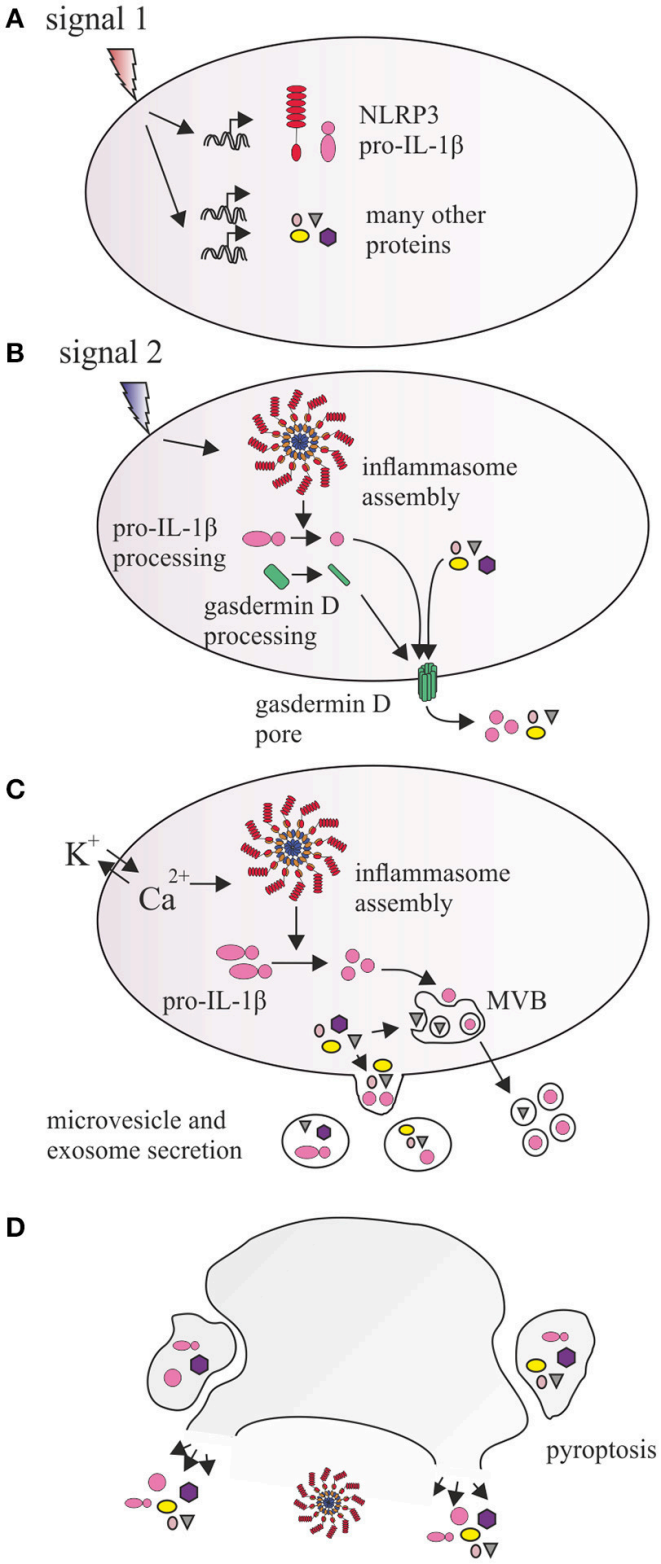
Proposed relationship between NLRP3 inflammasome and vesicular protein secretion. **(A)** Signal 1, provided by IL1R, TLRs, and other PRRs induces the transcription and expression of NLRP3, as well as precursor forms of inflammatory cytokines IL-1β and IL-18. It also leads to induction of expression of hundreds of other proteins. **(B)** Signal 2 activates inflammasome which catalyzes processing of pro-IL-1β and gasdermin D. N-terminal fragment of gasdermin D inserts into cell membrane and oligomerizes, forming a pore, which allows for direct secretion of small proteins, cytokines, and enhances ionic fluxes across the membrane. Additionally, calcium and potassium concentrations are also affected by membrane antiporter receptors (e.g., P2X7) or lysosomal leakage, amplifying inflammasome activation and causing activation of floppases, flippases, and scramblases, which catalyze transbilayer translocation of phosphatidylserine and phosphatidylethanolamine. **(C)** This affects membrane curvature, causing outward budding and formation of microvesicles, and incorporation of proteins in the vicinity of the membrane (including pro-IL-1β and mature IL-1β) as well as integral membrane proteins in their lumen. Rapid processing of IL-1β may also result in recruitment of mature IL-1β into intraluminal vesicles of multivesicular body (MVB), a component of endosomal pathway, which traffics its content directly to the membrane, releasing exosomes. **(D)** Prolonged inflammasome activity leads to pyroptosis which may involve release of inflammasome components, cytokines, and other proteins via membrane lysis or larger vesicular structures resulting from cell fragmentation.

Based on the available data, we postulate a model of inflammasome-dependent EV secretion as a sequential process, which serves the release of pro-inflammatory cytokines, as well as numerous other proteins whose relationship to inflammasome remains to be determined (Figure 1). Signal 1, delivered by surface PRRs during priming induces expression of inflammasome components and pro-inflammatory cytokines, but activates no inflammasome assembly or significant EV secretion [(25), Figure 1A]. Upon signal 2, assembly of the inflammasome with caspase-1 activation triggers processing of pro-inflammatory cytokines and gasdermin D. Active gasdermin D translocates to the membrane forming pores for cytokine secretion, but also further affecting transmembrane calcium/potassium equilibrium (Figure 1B). Cationic fluxes amplify signal 2 and simultaneously affect two parallel processes that facilitate formation and secretion of EV: (a) activating inflammatory caspases (possibly including non-canonical caspases 4 and 5) that are directly or indirectly involved in processing cytoskeletal proteins, compromising membrane rigidity and microvesicle formation, (b) modulating reorganization of plasma membrane sheets, with phosphatidylserine exposure as a result of calcium-activated phospholipid transporters. The forming shedding compartment carries the local cargo proteins, including processed and unprocessed cytokines, or even entire inflammasome complexes. Mature cytokines may also be targeted to multivesicular bodies (MVB) and secreted in exosomes. Thus at this stage, formation of EV via multiple mechanisms may postpone imminent cell death by removal of active inflammasome, and enhance the secretion of IL-1β (Figure 1C). During prolonged exposure to signal 2, the cell undergoes pyroptotic cell death, which is associated with membrane damage and leakage of cytoplasm contents. However, several studies suggested existence of caspase-1-dependent cell death that resembles apoptosis that may be activated concomitantly to pyroptosis or at later stages (74–77). Indeed *Gsdmd* deletion causes a shift from pyroptotic to apoptotic-like phenotype in mouse models (31). Similarly to classical apoptosis, such process may be associated with compartmentalization of cellular content and effective formation of EV similar to apopotic bodies (Figure 1D). Current knowledge about these “pyroptotic vesicles” is very limited, however their release has been showed in our recent study (17). Consequently, EV-mediated secretion may be a major inflammasome-dependent secretory route of IL-1β, IL-18, and other proteins important for coordination of immune response. There are however several questions still seeking answers. First—do all NLRP3 inflammasome activators enhance secretion of EV? Currently only a handful of canonical and one non-canonical NLRP3 stimulus have been directly shown to induce EV secretion. It has also not been determined whether EV secretion occurs via one preferred route, dependent possibly on a particular signal, or alternatively involving exosomes, shedding microvesicles and blebs to a similar extent. Second—it is obvious that kinetics of EV release varies significantly and may range from minutes (ATP) to hours (β-glucan) after inflammasome activation (14, 15). In order to corroborate the view of inflammasome-activated EV secretion, it is important to further investigate the patterns and kinetics of “weak” and “strong” NLRP3 activators (36) in stimulating EV secretion. Finally, it will be essential to dissect the signal transduction pathways activated by different inflammasome stimuli on one hand, and those downstream NLRP3 and caspases-4/5 on the other, monitoring their roles in formulation, cargo selection and secretion of EV. These investigations may in the future provide novel explanations on inflammasome and EV biology improving understanding of inflammatory diseases and revealing novel therapeutic targets.

## Author contributions

WC and SM designed and wrote the manuscript. TN critically revised the manuscript.

### Conflict of interest statement

The authors declare that the research was conducted in the absence of any commercial or financial relationships that could be construed as a potential conflict of interest.
